# ALKBH5 facilitates the progression of skin cutaneous melanoma via mediating ABCA1 demethylation and modulating autophagy in an m^6^A-dependent manner

**DOI:** 10.7150/ijbs.92994

**Published:** 2024-02-25

**Authors:** Hanwen Wang, Shixin Zhao, Hengdeng Liu, Yiling Liu, Zihui Zhang, Ziheng Zhou, Peng Wang, Shaohai Qi, Julin Xie

**Affiliations:** Department of Burn and Wound Repair Surgery, The First Affiliated Hospital of Sun Yat-sen University, Guangzhou, Guangdong, 510080, People's Republic of China.

**Keywords:** Skin cutaneous melanoma, N^6^-methyladenosine methylation, Alkylation repair homolog protein 5, ATP-binding cassette transporter A1, Autophagy

## Abstract

**Background:** N6-methyladenosine (m^6^A) is the most common and abundant mRNA modification, playing an essential role in biological processes and tumor development. However, the role of m^6^A methylation in skin cutaneous melanoma (SKCM) is not yet clear. This study analyzed the expression of m^6^A-related functional genes in SKCM and aimed to explore the key demethylase ALKBH5 mediated m^6^A modification and its potential mechanism in human SKCM.

**Methods:** Based on public databases, the m^6^A-related gene expression landscape in SKCM was portrayed. MeRIP-Seq and RNA-Seq were used to recognize the downstream target of ALKBH5. *In vivo* and *in vitro* functional phenotype and rescue functional experiments were performed to explore the mechanism of the ALKBH5-m^6^A-ABCA1 axis in SKCM.

**Results:** We found ALKBH5 upregulated in SKCM, associated with poor prognosis. ALKBH5 can promote melanoma cell proliferation, colony formation, migration, and invasion and inhibit autophagy *in vitro*, facilitating tumor growth and metastasis *in vivo*. We identified ABCA1, a membrane protein that assists cholesterol efflux, as a downstream target of ALKBH5-mediated m^6^A demethylation. Finally, our data demonstrated that ALKBH5 promoted SKCM via mediating ABCA1 downregulation by reducing ABCA1 mRNA stability in an m^6^A-dependent manner.

**Conclusion:** Our findings exhibited the functional value of the key demethylase ALKBH5 mediated m^6^A modification in the progression of SKCM, suggesting the ALKBH5-m^6^A-ABCA1 axis as a potential therapeutic target in SKCM.

## Introduction

Skin cutaneous melanoma (SKCM), the most common human skin malignancy, causes 55500 deaths and 90% of skin cancer mortality annually^1^-^3^. Early detection and surgical excision could often be curative in most cases. However, melanoma becomes life-threatening once a distant spread happens. Although immune checkpoint inhibitors targeting PD1/PDL1, and clinical applications of BRAF and MEK inhibitors have revolutionized the treatment of SKCM in the last decade, long-term survival for metastatic melanoma is still low^4^-^7^. Management of novel target interventions may help prevent tumor metastasis, improving clinical outcomes. Herein, we investigated the mechanisms of SKCM progression, providing a more extensive vision of the biology of cancer.

N^6^-methyladenosine (m^6^A) is the most common mRNA modification in eukaryotic biological activities^8^, ^9^. As a dynamically reversible internal mRNA modification, its acquisition and removal are regulated by m^6^A "writer" proteins (Methyltransferase-like 3 [METTL3], METTL14, Wilms tumor 1 associated protein [WTAP], and VIRMA [KIAA1429]), m^6^A "eraser" proteins (Fat-mass and obesity-associated protein [FTO] and Alkylation repair homolog protein 5 [ALKBH5]), and specific "reader" proteins (YTH domain-containing proteins, YTHDF1/2/3, YTHDC1/2, and Human insulin-like growth factor 2 mRNA binding proteins, IGF2BP1/2/3), which recognize m^6^A modification sites and change mRNA status including mRNA stability, decay, splicing, and translation^10^, ^11^. Recent studies have demonstrated that abnormal statuses of m^6^A methylation can participate in tumor proliferation, metastasis, metabolism, and drug resistance, and are closely related to the occurrence and progression of various malignant tumors^12^-^15^. Nevertheless, the function and mechanism of m^6^A methylation in the malignant progression of SKCM remain unclear.

In the present study, we portraited the landscape of expression profiles of m^6^A-related genes in SKCM and discovered that m^6^A demethylase ALKBH5 was up-regulated, associated with poor prognosis. Furthermore, we explored the effect of ALKBH5 in SKCM by *in vivo* and *in vitro* experiments and the underlying regulatory mechanism of the m^6^A modification mediated by ALKBH5 in SKCM, proposing a novel prognostic predictor and therapeutic target for SKCM progression.

## Materials and Methods

### Patient samples, cell lines and cell culture

A total of 18 SKCM and 8 normal skin (NS) paraffin-embedded specimens were collected from the Department of Pathology of the First Affiliated Hospital of Sun Yat-sen University. The experiments and sample collection followed the Declaration of Helsinki and were approved by the Medical Ethics Committee of the First Affiliated Hospital of Sun Yat-sen University.

SKCM cell lines A375 and A2058 were obtained from American Type Culture Collection (ATCC) with authentication. These cell lines were cultured in Dulbecco's modified Eagle's medium (DMEM, Corning, USA) with 10% fetal bovine serum (Gibco, USA) and antibiotics (Servicebio, China). Cells were grown in a 5% CO_2_ cell culture incubator at 37 °C.

### Screening of the differentially expressed m^6^A RNA methylation related genes

Totally 461 SKCM samples from The Cancer Genome Atlas (TCGA) and 558 normal skin (NS) samples from the GTEx database are acquired and included for screening, and |log2FC|>0.5 and *p*-value < 0.05 are regarded as differentially expressed. The UALCAN database was used to compare the expression differences between primary tumors and metastatic tumors.

### Immunohistochemistry and immunocytofluorescence

For immunohistochemistry (IHC) analysis, tissue slides were deparaffinized and rehydrated through an alcohol series followed by antigen retrieval with sodium citrate buffer, blocked with 5% normal goat serum (Vector) with 0.1% Triton X-100 and 3% H_2_O_2_ in PBS for 60 min at room temperature and then incubated with appropriate primary antibodies against ALKBH5(1:1000, ab195377, Abcam), ABCA1(1:200, ab18180, Abcam), and Ki67 (1:1000, ab15580, Abcam) at 4 °C overnight. IHC staining was performed with horseradish peroxidase (HRP) conjugates using DAB detection. Nuclei were counterstained with Hoechst.

For immunocytofluorescence (IF), SKCM cells were first treated with 4% PFA for 15 min. The sections and cells were blocked with Immunol Staining Blocking Buffer (Beyotime) for 1 h and incubated with the primary antibody against ABCA1(1:200, ab18180, Abcam) overnight at 4 °C, then were incubated with Alexa Fluor secondary antibodies (Jackson ImmunoResearch, PA, USA) for 1 h. This was followed by incubation with a diamidinyl phenyl indole (DAPI) coloration for 10 min (Servicebio, China). Images were taken with the fluorescence microscope (IX83, Olympus, Japan).

### LC-MS analysis of RNA m^6^A level

RNA from A375 cell lines was extracted with TRIzol (ThermoFisher, USA). The integrity and quantity of each sample were examined using agarose gel electrophoresis and Nanodrop™ instrument. The mRNA was isolated and purified from total RNA using NEBNext® Poly(A) mRNA Magnetic Isolation Module and then hydrolyzed to single nucleosides. Nucleosides were further dephosphorylated by enzyme mix. The pretreated nucleoside solution was deproteinized using the Sartorius 10000-Da MWCO spin filter.

Nucleoside mixtures were analyzed on Agilent 6460 QQQ mass spectrometer with an Agilent 1290 HPLC system. Multi-reaction monitoring (MRM) mode was performed because of its high selectivity and sensitivity attained working with parent-to-product ion transitions. LC-MS data was acquired using Agilent Qualitative Analysis software. MRM peaks of each modified nucleoside were extracted and normalized to peak areas of normal adenosine in each sample. Samples were run in duplicate, and m^6^A/A ratios were calculated.

### Plasmid constructions and lentiviral transfection

Small hairpin RNAs (shRNAs) of ALKBH5 and ABCA1 were obtained from VectorBuilder (Guangzhou, China), and then annealed into the pLKO·1-puro vector to construct the knockdown plasmids pLKO·1-ALKBH5 and pLKO·1-ABCA1 vectors. All of the shRNA sequences are listed in **Table S1**.

2×10^5^ SKCM cells were added to each well of the 6-well plate with lentiviruses at the concentration of MOI=10, 1 ml DMEM medium, and 1:1000 polybrene (VectorBuilder, Guangzhou, China). After culturing for 12 h, the cells were washed twice with PBS and replaced with fresh DMEM. After another 24h, the cells were replaced with DMEM containing puromycin and screened for 3 days.

### RNA isolation and RT-qPCR

Total RNA was extracted by TRIzol (Invitrogen, USA) following the manufacturer's instructions. Complementary DNA (cDNA) synthesis was performed with GeneAmp RNA PCR kit (Invitrogen) using 1 µg RNA per sample. Real-time reverse transcription fluorescent quantitative polymerase chain reaction (RT-qPCR) was performed using the Real-time Fluorescent Quantitative PCR Kit (Vazyme, Q321) and an ABI7900HT Fast Real-Time PCR system (Applied Biosystems, CA, USA). GAPDH was used as an endogenous control. The comparative Ct(2^-△△CT^) method was applied to calculate the relative expression. All primers used in this study are listed in **Table S2**.

### Western blotting

Cells were lysed in RIPA buffer (P0013B, Beyotime, China) containing the complete cocktail of protease inhibitors (#11836153001, Roche, Switzerland). Protein concentrations were determined with the BCA protein assay kit (P0011, Beyotime, China). Proteins were separated by 4-20% SDS-PAGE, transferred to PVDF film and blotted with antibodies at 4 °C overnight. Secondary antibodies were pre-labelled at room temperature for 1 h. The PVDF film with the target protein was exposed in the visualizer (AI800, GE, USA). Antibodies were purchased from the following: ALKBH5 (ab195377, 1:1000, Abcam), Caspase-3 (A19654, 1:1000, Abclonal), SQSTM1/p62 (A19700, 1:1000, Abclonal), GAPDH (A19056, 1:10000, Abclonal).

### *In vitro* cell proliferation, colony formation, migration, invasion and apoptosis assay

For cell proliferation assay, SKCM cells were seeded in 96-well microplates at a density of 3000 cells per well, and cell viability was measured using Cell Counting Kit-8 (Biosharp, China) at the time in 0h, 24h, 48h, 72h, and 96h. The microplates were incubated at 37 °C for an additional 2 hours. Absorbance was read at 450 nm using a microplate reader (ThermoFisher, USA).

For colony formation assay, SKCM cells were seeded into 6-well plates at a density of 500 cells per well. Following 1 week of incubation, the cell colonies were fixed with 4%PFA and stained with crystal violet.

For transwell migration and invasion assays, 3×10^4^ cells were suspended in 100μl serum-free medium and seeded in the upper chamber (the floor of the upper chamber was coated with matrigel (1:10 dilution, Corning, USA) in the invasion assay). The lower chamber was filled with 600μl medium containing 10% fetal bovine serum to induce migration and invasion. After 24h of incubation at 37 °C, the cells on the upper surface of the membrane were removed. Cells attached to the lower surface were fixed by 4% PFA and stained using 0.5% crystal violet for 20 min. The view of migrating and invading cells was imaged and counted using a light microscope (Olympus, Japan).

For cell apoptosis assay, the Annexin V-PE/7AAD Apoptosis Detection Kit (559763, BD, USA) was used to stain the collected SKCM cells following the manufacturer's instructions, and the cells were analyzed with a flow cytometer (Celesta, BD, USA).

### Animal experiments

Nude mice were all housed under pathogen-free conditions. All animal care and experiments were approved by the Institutional Animal Care and Use Committee of Sun Yat-sen University, and the study is compliant with all relevant ethical regulations regarding animal research. Mice were euthanized when they met the institutional euthanasia criteria for tumor size and overall health condition.

For the subcutaneous implantation model, 6 four-week-old female nude mice were injected with 5 × 10^6^ A375 cells in 100μl of mixed medium (50ul serum-free DMEM and 50ul matrigel) at the right flank of the back. Tumors were measured with a calliper every 3 days to analyze tumor growth. Tumor volume was calculated by the formula: V = (width^2^ × length)/2. At the experimental endpoint, tumor tissues were harvested and fixed with 4% PFA for the paraffin-embedded section.

For tumor metastasis mice model, 6 four-week-old female mice were randomly grouped and injected with 1 × 10^6^ A375 cells in 100ul PBS via the tail vein. Mice were sacrificed 4 weeks after injection to detect lung metastasis. Lung tissues were harvested and fixed with 4% PFA for the paraffin-embedded section, and lung metastasis was detected with microscopy.

### RNA-Seq

The A375 cells, infected with lentiviruses expressing Scramble and shALKBH5, were harvested at 48 h post-infection, followed by RNA extraction using TRIzol (Invitrogen, USA). The cDNA library was prepared by LC-Bio (Hangzhou, China). The paired-end reads were generated by the Illumina® HiSeq 2500 platform supplied by LC-Bio. An R package, EdgeR, was used to quantify transcription levels and identify differentially expressed genes, using a cut-off of *p*-value < 0.05.

### MeRIP-Seq and MeRIP-qPCR

Total RNA was isolated and purified using TRIzol reagent (Invitrogen, USA) from indicated A375 cells. The RNA amount and purity of each sample was quantified using NanoDrop ND-1000 (NanoDrop, USA), and the RNA integrity was assessed by Bioanalyzer 2100 (Agilent, USA). The mRNA was further purified using Dynabeads mRNA Purification Kit (61006, Invitrogen, USA). After fragmentation with Magnesium RNA Fragmentation Module (NEB, USA), the anti-m^6^A antibody (202003, Synaptic Systems, Germany) was used for immunoprecipitated. The IP RNA was reverse-transcribed to cDNA by SuperScript™ II Reverse Transcriptase (Invitrogen, USA), which was next used to synthesise U-labeled second-stranded DNAs with *E. coli* DNA polymerase I (NEB, USA), RNase H (NEB, USA) and dUTP Solution (Thermo Fisher, USA). At last, we performed paired-end sequencing (PE150) on an Illumina Novaseq™ 6000 platform (LC-Bio, Hangzhou, China) or MeRIP-qPCR analysis.

### Luciferase reporter assay

The 3'UTR regions of ABCA1 were amplified by PCR from cDNA and cloned into the vector to construct WT-ABCA1 dual-luciferase reporter plasmid. The 3'UTR fragment, which mutated at the m^6^A site of ABCA1 with thymidine (T) to adenosine (A), was synthesized by GeneChem (Shanghai, China) and cloned into the vector to construct Mut-ABCA1 dual-luciferase reporter plasmid. Luciferase activity was measured by Dual-Luciferase Reporter Gene Assay Kit (RG028, Beyotime, China) in GM2000 (Promega). Experiments were performed using 293T cells in triplicates. The firefly luciferase activity values were normalized to the Renilla luciferase activity values that reflect expression level.

### RNA decay assay

Cells were treated with actinomycin D (Sigma, USA) for 0 h, 3 h, and 6 h, followed by cellular RNA extraction, and the half-life of ABCA1 mRNA was analyzed by RT-qPCR.

### Statistical analysis

Statistical analysis was performed using GraphPad Prism 8.0. Each experiment was repeated three times independently. Data are presented as the mean ± standard deviation. Two-tailed Student's t-test was used to compare the statistical difference between indicated groups. Overall survival was analyzed by the Kaplan-Meier and log-rank tests. Univariate and multivariate Cox regression models were used to study independent prognostic factors. Statistical significance was accepted for *p*-value < 0.05.

## Results

### Demethylase ALKBH5 is upregulated in SKCM and related to poorer survival

**Figure 1:**
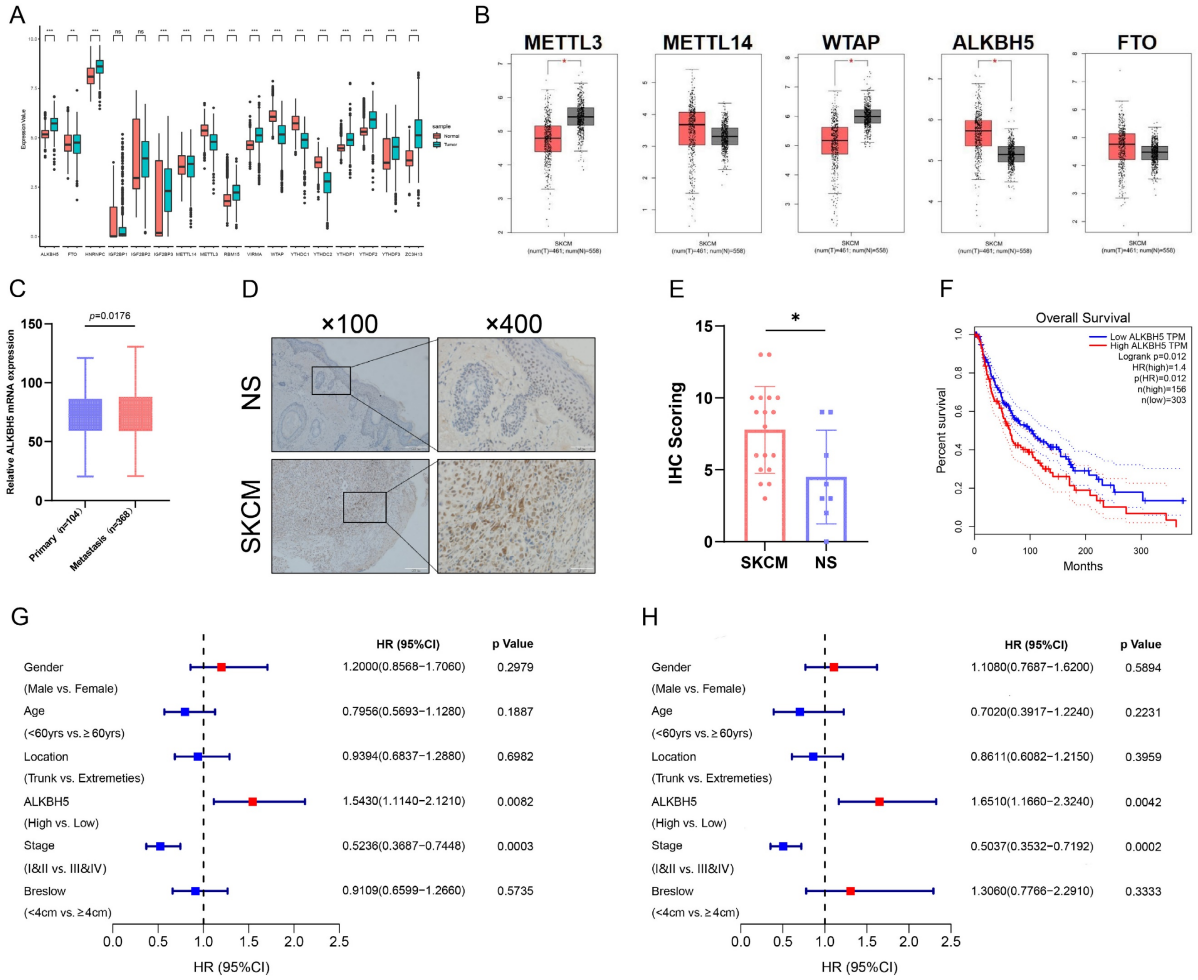
** Demethylase ALKBH5 is upregulated in SKCM and related to poorer survival. (A)** The expression landscape of m^6^A-related genes in SKCM and NS. **p-value < 0.01, ***p-value < 0.001.** (B)** The differential expression analysis of major m^6^A writers and erasers between SKCM and NS. |log2FC|>0.5, *p-value < 0.05. **(C)** The relative ALKBH5 mRNA level between primary and metastatic melanoma.** (D)** The higher protein level of ALKBH5 in SKCM tissues than in NS tissues was verified by immunohistochemistry assay. **(E)** Statistical analysis results of immunohistochemistry.** (F)** Kaplan-Meier survival analysis displayed that the overall survival time of patients with high ALKBH5 expression was significantly shorter than that of patients with low ALKBH5 expression (log-rank *p* = 0.012).** (G)** Univariate analysis showed that the high level of ALKBH5 was a risk factor for SKCM (*p* = 0.0082).** (H)** Multivariable analysis showed that the high level of ALKBH5 was a risk factor for SKCM (*p* = 0.0042).

To acquire the insight into the potential m^6^A modification in SKCM progression, we analyzed the transcriptomic profiles of SKCM tissue from the TCGA and normal skin (NS) tissue from the GTEx database. We detected relative expression levels of m^6^A-related genes between SKCM and NS and identified that the core m^6^A demethylase ALKBH5 was significantly up-regulated in SKCM tissue (**Figure 1A and 1B**). Through the UALCAN tool, we further discovered that the expression level of ALKBH5 was significantly higher in metastatic melanoma than in primary melanoma, indicating that ALKBH5 may play a dominant part in cancer progression and metastasis in SKCM (**Figure 1C**). Subsequently, we validated the high expression of ALKBH5 in SKCM rather than in NS samples through immunohistochemistry staining, which supported our initial analysis (**Figure 1D and 1E**). Next, we investigated the prognostic value of ALKBH5 in the SKCM cohort from the TCGA database. The Kaplan-Meier analysis was applied, and SKCM patients with high ALKBH5 expression held poorer OS (*P* = 0.012) (**Figure 1F**). Univariate regression analysis demonstrated that advanced tumor stages (III & IV) and high expression of ALKBH5 were both hazard factors of OS (**Figure 1G**). Meanwhile, Multivariate regression analysis also indicated that ALKBH5 expression was an independent predictor (HR = 1.651, 95% CI [1.166-2.324]) of OS for the cohort (**Figure 1H**). These outcomes implied that ALKBH5 was overexpressed in SKCM and might be an independent prognostic signature for SKCM patients.

### ALKBH5 promoted SKCM cell proliferation, colony formation, migration, invasion and suppressed autophagy *in vitro*

**Figure 2:**
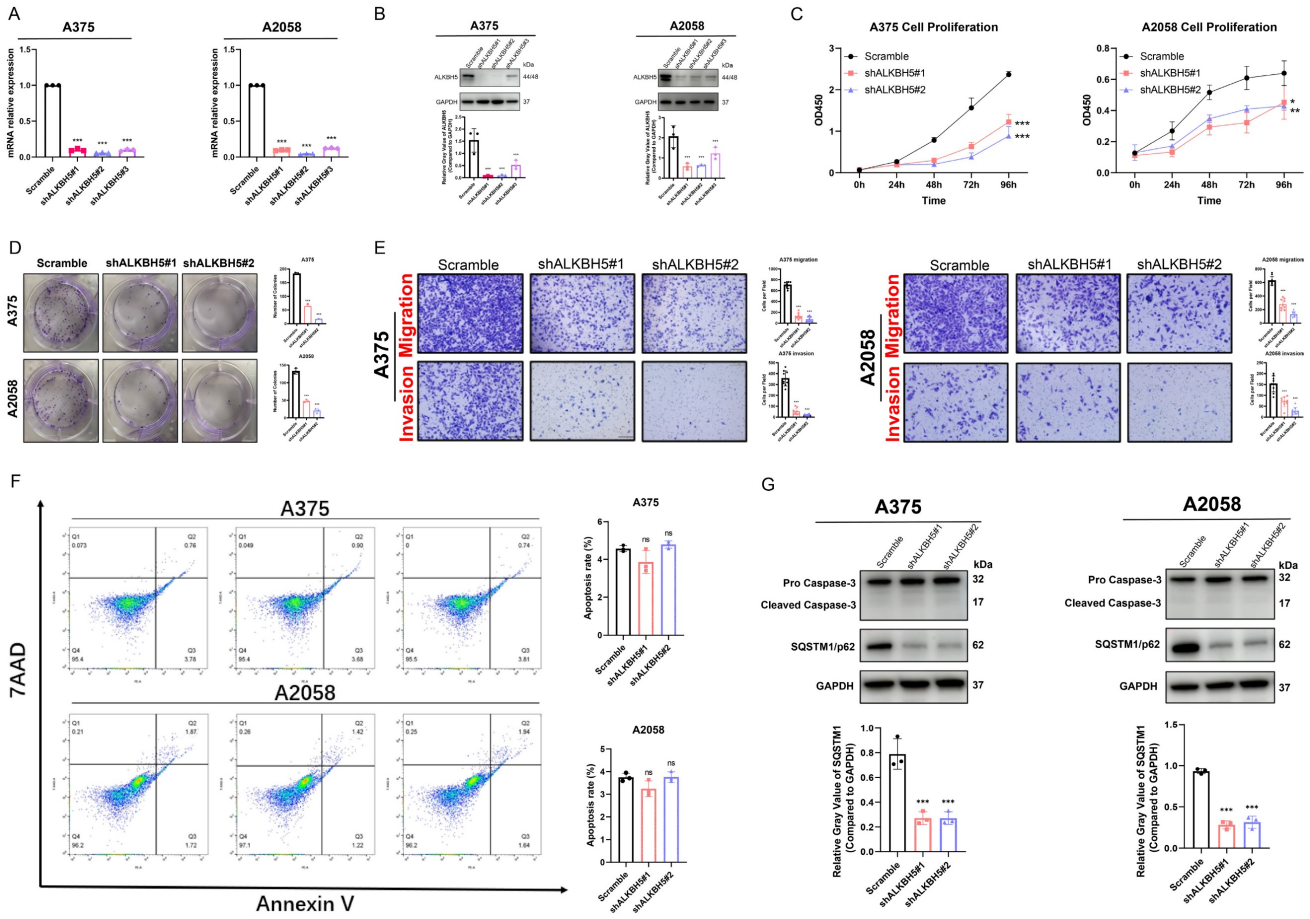
** ALKBH5 promoted SKCM cell proliferation, colony formation, migration, invasion, and suppressed autophagy *in vitro*. (A)** RT-qPCR confirmed the knockdown of ALKBH5 in A375 and A2058 cells by shRNA on mRNA levels.** (B)** Western blotting confirmed the knockdown of ALKBH5 in A375 and A2058 cells by shRNA on protein levels. **(C)** Cell growth curve of A375 and A2058 cells transfected with Scramble or shALKBH5. **(D)** Colony formation ability of A375 and A2058 cells transfected with Scramble or shALKBH5. **(E)** The apoptosis rate of A375 and A2058 cells transfected with Scramble or shALKBH5. **(F)** Cell migration and invasion ability of A375 and A2058 cells transfected with Scramble or shALKBH5. **(G)** The protein levels of ALKBH5 and SQSTM1 in A375 and A2058 cells transfected with Scramble or shALKBH5. The results are presented as mean ± SD. * p < 0.05, ** p < 0.01, *** p < 0.001.

To investigate the specific function of ALKBH5 in the progression of SKCM, we transfected cells with lentiviruses packaged with distinct shRNA sequence plasmids (shALKBH5#1, shALKBH5#2, and shALKBH5#3) and constructed stable ALKBH5-knockdown cells in melanoma A375 and A2058 cell lines. The knockdown efficiency of ALKBH5 was verified by both RT-qPCR and western blotting (**Figure 2A and 2B**). As shown in CCK-8 and colony formation assay, the proliferation and colony formation ability of A375 and A2058 cells was significantly reduced after the knockdown of ALKBH5 (**Figure 2C and 2D**). The transwell assay was then performed, and the consequences suggested that the knockdown of ALKBH5 inhibited the migration and invasion of SKCM cells (**Figure 2E**). We next used flow cytometry with Annexin V/7AAD staining to analyze the influence of ALKBH5 in cell apoptosis. ALKBH5 depletion did not significantly affect cell apoptosis rate in either A375 or A2058 cells (**Figure 2F**). The western blotting results of apoptosis-related signature Caspase-3 also approved this standpoint. In addition, we performed western blotting on the autophagy substrate Sequestosome 1 (SQSTM1/p62). We found that knocking down ALKBH5 resulted in a decrease in SQSTM1 protein expression, which was negatively correlated with the degree of cell autophagy, promoting autophagy in A375 and A2058 melanoma cells (**Figure 2G**). Overall, our results presented that ALKBH5 was essential in assisting SKCM cell growth, colony formation, migration, invasion, and inhibiting cell autophagy.

### Inhibition of ALKBH5 suppressed SKCM growth and metastasis *in vivo*

**Figure 3:**
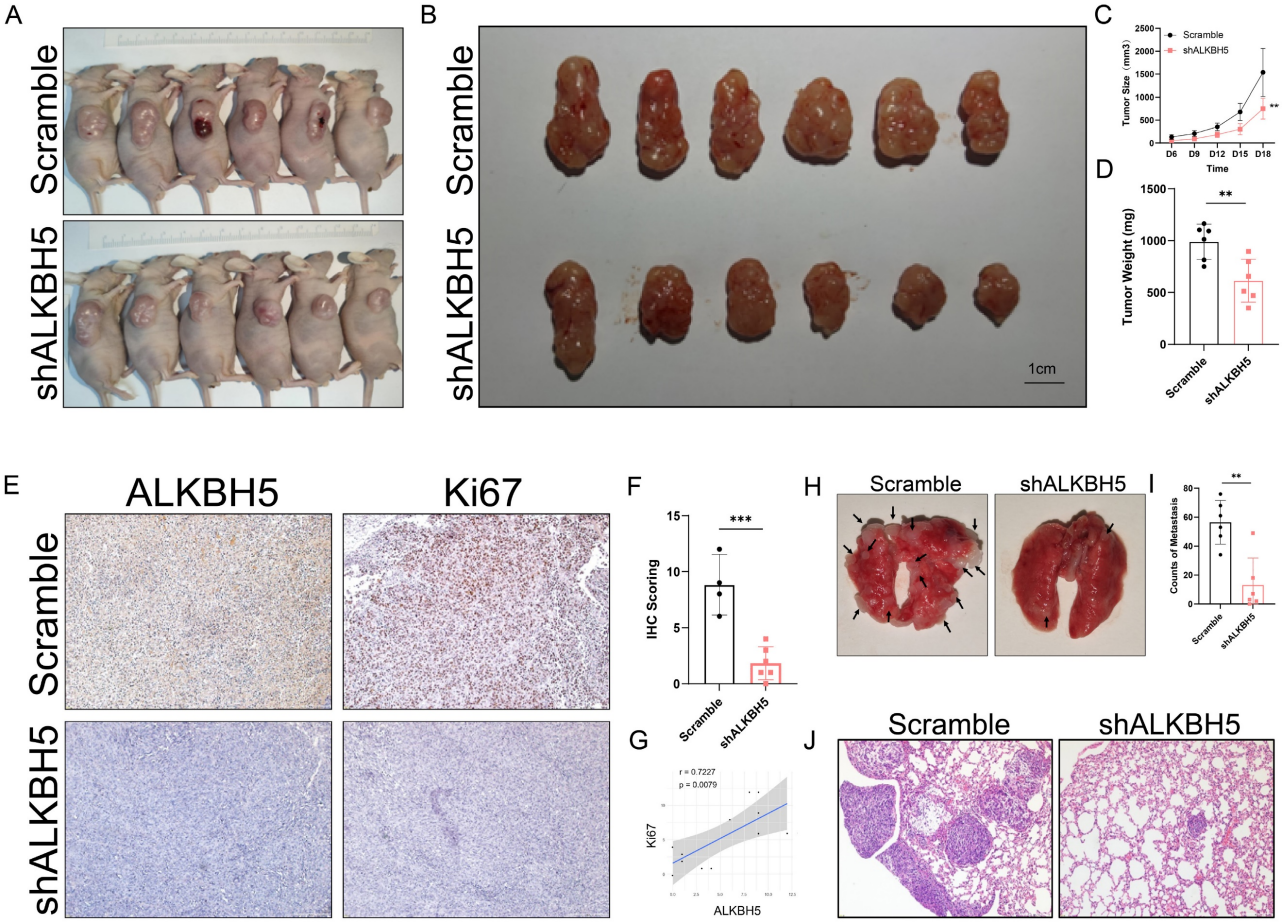
** Inhibition of ALKBH5 suppressed SKCM growth and metastasis *in vivo*. (A)** General observation of nude mice in tumor subcutaneous implantation experiment. **(B)** General observation of tumor with A375 cells transfected with shALKBH5 or Scramble.** (C)** The size of the subcutaneous implanted tumor was measured every three days. **(D)** The weight of the subcutaneous implanted tumor was measured at the end. **(E)** Images of IHC staining showing ALKBH5, and Ki67 in subcutaneous tumor models. **(F)** Statistical analysis results of immunohistochemistry staining of Ki67.** (G)** Correlation analysis of ALKBH5 and Ki67 protein expression levels in immunohistochemistry staining. **(H)** General observation of lung tissues of the nude mice treated with the indicated cells. **(I)** Counting of pulmonary metastatic nodules at the end. **(J)** The pulmonary metastatic nodules were verified by hematoxylin and eosin staining. The results are presented as mean ± SD. * p < 0.05, ** p < 0.01, *** p < 0.001.

To investigate the function of ALKBH5 in the growth and metastasis of SKCM, we conducted *in vivo* experiments with stable ALKBH5-knockdown A375 cells in nude mice. As shown in the consequence of the tumor subcutaneous implantation experiment, silencing of ALKBH5 effectively repressed tumor growth (**Figure 3A and 3B**), as reflected by the significant decline of tumor size and weight compared to the negative control group (**Figure 3C and 3D**). We also observed from the immuno-histological analysis that a decrease in the expression of Ki67, a cell proliferation marker, accompanied the knockdown of ALKBH5 (**Figure 3E-3G**). Furthermore, we established SKCM lung metastasis models in nude mice through tail vein injection (**Figure 3H**). When the expression of ALKBH5 was downregulated, the number of pulmonary metastatic nodules was also reduced (**Figure 3I and 3J**). All these results suggest that ALKBH5 and its targets are essential in facilitating SKCM progression and metastasis *in vivo*.

### Identification of potential downstream targets of ALKBH5 in SKCM

**Figure 4:**
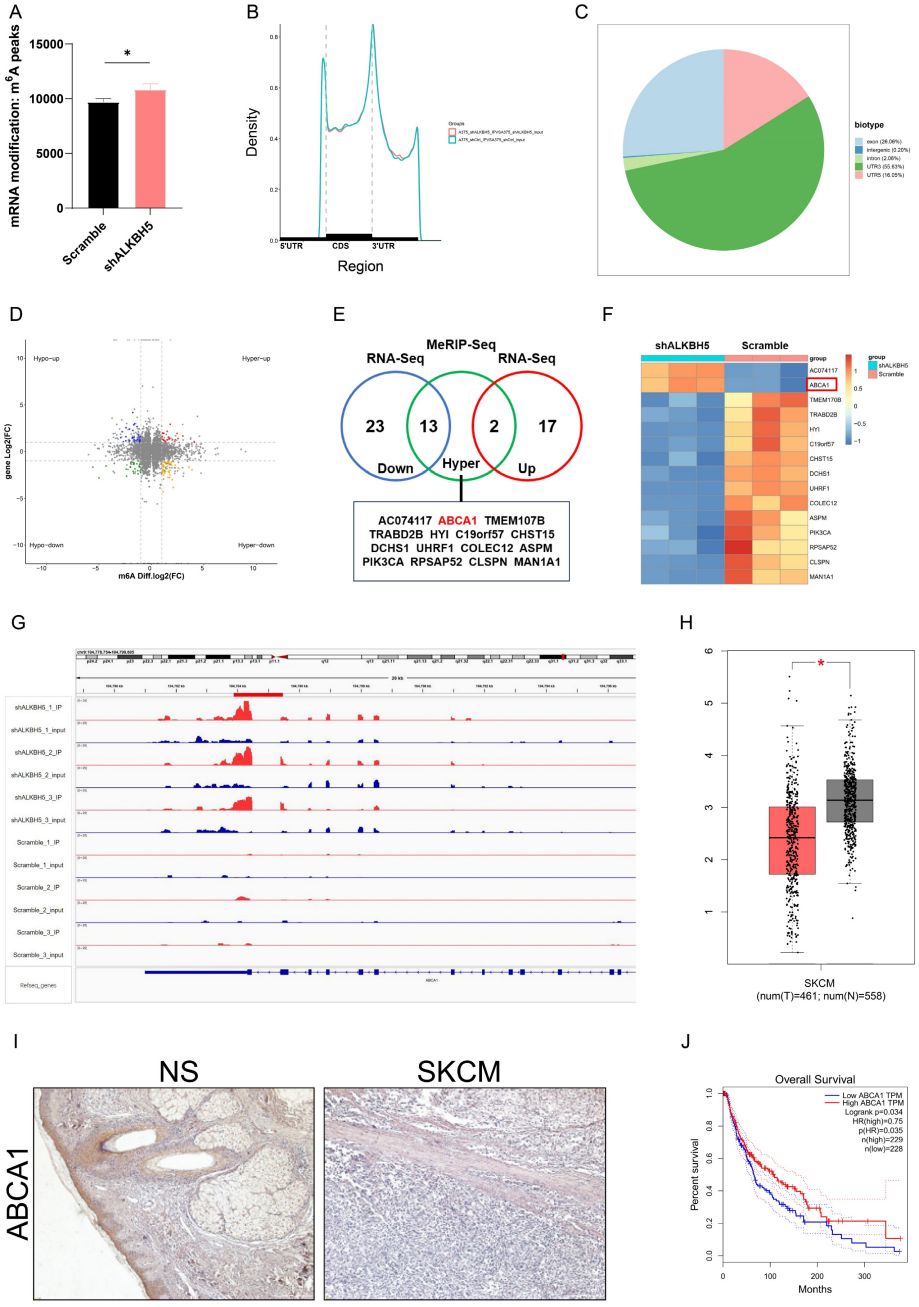
**Identification of ABCA1 as a potential downstream target of ALKBH5 in SKCM. (A)** SKCM A375 cells transfected with Scramble exhibited lower total m^6^A methylation levels compared to the shALKBH5 group. **(B)** Divide genes into three regions: 5'UTR, CDS, and 3'UTR, and calculate the distribution of peaks in each area. **(C)** Peaks are mostly enriched in the 3'UTR region. **(D)** Volcano plots for RNA-Seq and MeRIP-Seq results of ALKBH5-knockdown A375 cells versus controls. Significantly hypermethylated-upregulated (red), hypermethylated-downregulated (yellow), hypomethylated-upregulated (blue), and hypomethylated-downregulated(green) genes are shown. **(E)** Venn diagram illustrated the overlap of significantly hypermethylated genes and differentially expressed genes. **(F)** Heatmap showed the hypermethylated differentially expressed genes in ALKBH5-knockdown A375 and control cells. **(G)** IGV plot showed the increased m^6^A modification in ABCA1 mRNA in ALKBH5-knockdown A375 cells than controls. **(H)** The mRNA expression level of ABCA1 in SKCM is lower than in normal skin. |log2FC|>0.5, *p-value < 0.05. **(I)** The higher protein level of ABCA1 in NS tissues than in SKCM tissues was verified by immunohistochemistry assay.** (J)** Kaplan-Meier survival analysis displayed that the overall survival time of patients with high ABCA1 expression was significantly longer than that of patients with low ALKBH5 expression (log-rank *p* = 0.034).

LC-MS analysis showed that SKCM A375 cells in the shALKBH5 group had significantly more m^6^A peaks than the control group, indicating that ALKBH5 has epigenetic effects on SKCM cells (**Figure 4A**). To further explore the epigenetic mechanism underlying how ALKBH5 facilitates tumor progression and recognizes the downstream targets in SKCM, we performed RNA-Seq and MeRIP-Seq using shALKBH5 and negative control A375 cells. RNA-Seq analysis showed that compared to the control group, 36 genes (log2FC<-2) were downregulated, and 19 genes (log2FC>2) were upregulated when ALKBH5 was silenced (**Figure 4D and 4E**).

For MeRIP-Seq, the m^6^A signal was mainly enriched in 5'UTR, CDS, and 3'UTR. Besides, the differential m^6^A peaks were most frequently detected at 3'UTR (**Figure 4B and 4C**). To evaluate whether the altered gene expression was generated by m^6^A modification, we overlapped the increased m^6^A peaks (log2FC>1) of MeRIP-Seq and 55 differential genes of RNA-Seq, gaining 15 genes (**Figure 4E and 4F**). Among these genes, we found that ATP-binding cassette transporter A1 (ABCA1), a functional membrane protein facilitating cholesterol efflux, presented the most consistent raised m^6^A level after ALKBH5 knockdown, and its mRNA level increased in shALKBH5 A375 melanoma cells compared to control cells (**Figure 4G**). Differential expression analysis from databases suggested low expression of ABCA1 in SKCM compared to normal skin, and we applied immunohistochemistry to verify the low expression of ABCA1 in SKCM tissue (**Figure 4H and 4I**). Meanwhile, the Kaplan-Meier curve showed that a low expression level of ABCA1 was accompanied by a better prognosis, suggesting that ABCA1 may be a tumor suppressor gene, which accorded with the logic that upstream ALKBH5 plays as a potential oncogene (**Figure 4J**). Therefore, we chose ABCA1 as a potential downstream targeted gene after ALKBH5 demethylation for additional investigations.

### ALKBH5 mediated epigenetic silencing of ABCA1 in an m^6^A-dependent manner

**Figure 5:**
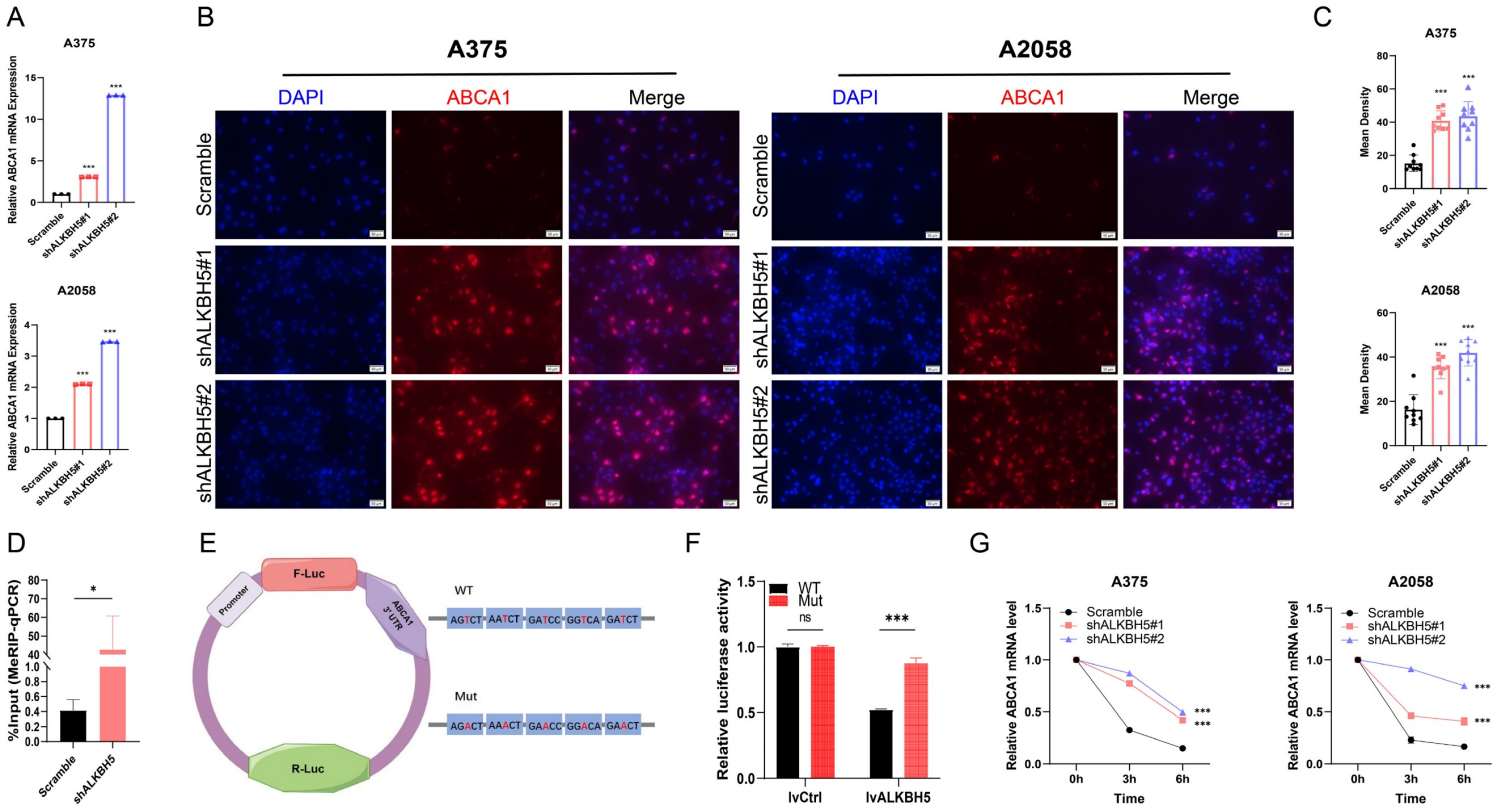
** ALKBH5 mediated epigenetic silencing of ABCA1 in an m^6^A-dependent manner. (A)** The results of RT-qPCR showed the ABCA1 mRNA expression in A375 and A2058 cells.** (B)** The images of immunocytofluorescence showing the protein expression of ABCA1 in A375 and A2058 cells.** (C)** The results of immunocytofluorescence showed that knocking down ALKBH5 led to increased protein expression of ABCA1 in A375 and A2058 cells.** (D)** MeRIP-qPCR analysis of the m^6^A level in A375 cells. **(E)** Wildtype or m^6^A consensus sequence mutant ABCA1 3'UTR was merged with firefly luciferase reporter. The mutation of m^6^A consensus sequences was developed by substituting thymidine in the potential m6A motifs with adenosine. **(F)** Relative luciferase activity of wildtype and mutant ABCA1 3'UTR reporter vectors in ALKBH5-overexpressed and control group 293T cells. **(G)** The mRNA stability of ABCA1 in A375 and A2058 cells with ALKBH5 silencing. The results are presented as mean ± SD. * p < 0.05, ** p < 0.01, *** p < 0.001.

To confirm ABCA1 as a target of ALKBH5-mediated demethylation, we detected mRNA and protein expression levels of ABCA1 in SKCM cells. In accordance with our RNA-Seq results, ABCA1 was significantly upregulated in stable ALKBH5-knockdown A375 and A2058 cells (**Figure 5A-5C**). To validate ABCA1 as a demethylation target site of ALKBH5, we performed the m^6^A-RNA immunoprecipitation assay and RT-qPCR. As anticipated, the knockdown of ALKBH5 remarkably elevated the m^6^A level of ABCA1 mRNA (**Figure 5D**).

The MeRIP-Seq identified five potential m^6^A motifs in the 3′ UTR region of ABCA1. To verify the function of m^6^A methylation in the regulation of ABCA1, we inserted the target region into the luciferase reporter and transformed thymidine (T) in the potential m^6^A motifs into adenosine (A) to construct the mutant one (**Figure 5E**). Relative normalized luciferase activities of the wild-type and mutant ABCA1 3'UTR reporter vectors were compared in the ALKBH5-overexpressed and control cells. As expected, transfecting the wild-type reporter but not the mutant reporter into ALKBH5-overexpressed cells reduced the luciferase activity (**Figure 5F**). In addition, the mRNA stability assay showed that the silencing of ALKBH5 lessened the degradation rate of ABCA1 mRNA, enhancing its stability (**Figure 5G**).

### Depletion of ABCA1 alleviated the suppression of SKCM by ALKBH5 inhibition *in vitro* and *in vivo*

**Figure 6:**
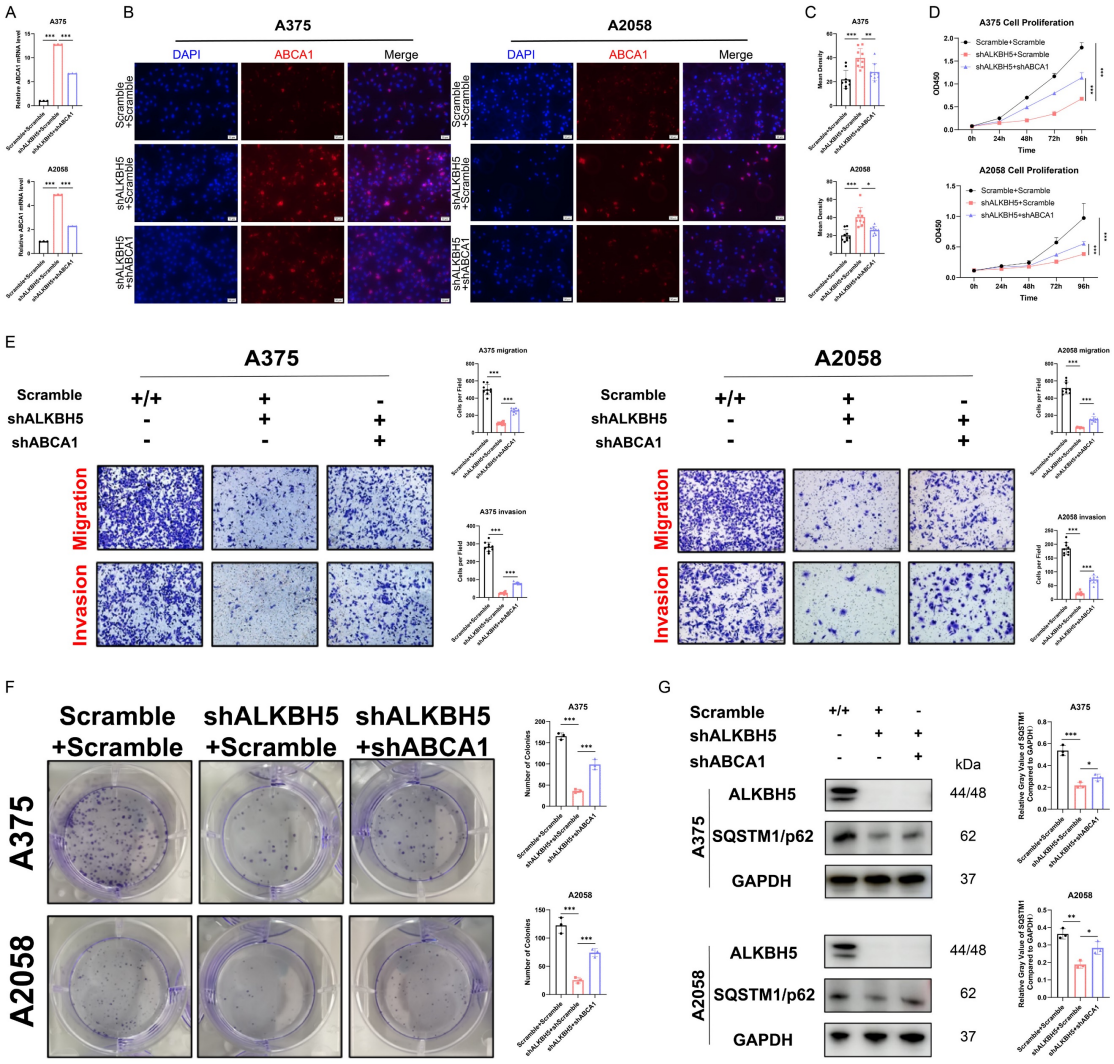
**Depletion of ABCA1 alleviated the suppression of SKCM by ALKBH5 inhibition *in vitro*. (A)** RT-qPCR confirmed the knockdown of ABCA1 in A375 and A2058 cells on mRNA level. **(B)** Immunocytofluorescence confirmed the knockdown of ABCA1 in A375 and A2058 cells on protein level. **(C)** The statistical results of immunocytofluorescence assay. **(D)** Cell growth curve of A375 and A2058 cells. **(E)** Cell migration and invasion ability of A375 and A2058 cells.** (F)** Colony formation ability of A375 and A2058 cells. **(G)** The protein levels of ALKBH5 and SQSTM1 in A375 and A2058 cells. The results are presented as mean ± SD. * p < 0.05, ** p < 0.01, *** p < 0.001.

To clarify whether ABCA1 participated in ALKBH5-dependent tumor progression and metastasis, we silenced ABCA1 in ALKBH5 stable knockdown SKCM A375 and A2058 cells (**Figure 6A-6C**). We found that the loss of ABCA1 alleviated the inhibition of cell proliferation, colony formation, migration, and invasion ability mediated by ALKBH5 (**Figure 6D-6F**). Meanwhile, the western blotting results also suggested that inhibition of ABCA1 leads to partially restoring autophagy substrate SQSTM1, which repressed cell autophagy generated by silencing ALKBH5 (**Figure 6G**).

**Figure 7:**
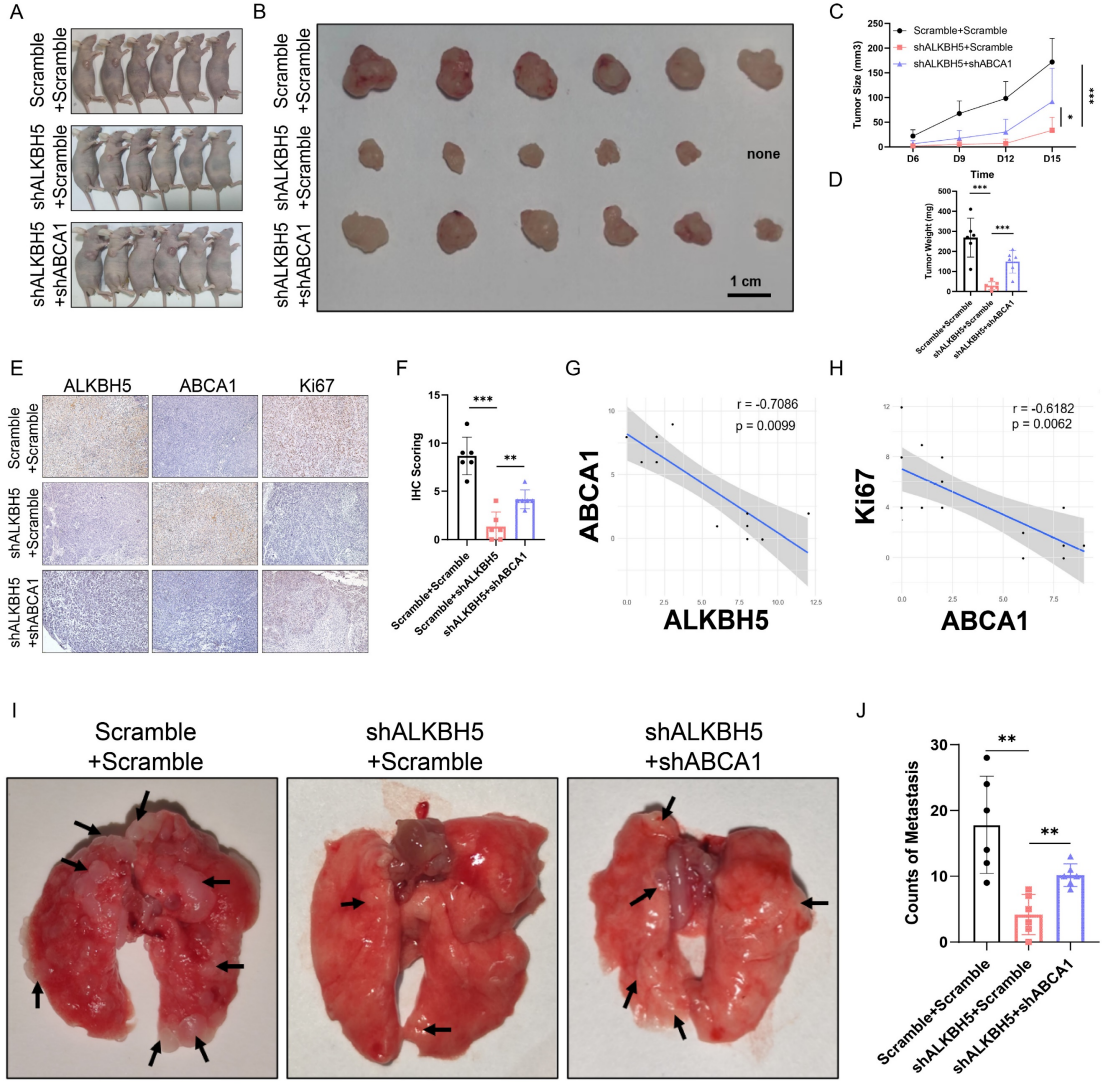
** Depletion of ABCA1 alleviated the suppression of SKCM by ALKBH5 inhibition *in vivo*. (A)** General observation of nude mice in tumor subcutaneous implantation experiment.** (B)** General observation of tumor with A375 cells transfected with ALKBH5 shRNA and/or ABCA1 shRNA.** (C)** The size of the subcutaneous implanted tumor was measured every three days. **(D)** The weight of the subcutaneous implanted tumor was measured at the end.** (E)** Images of IHC staining showing ALKBH5, ABCA1, and Ki67 in subcutaneous tumor models. **(F)** Statistical analysis results of immunohistochemistry staining of Ki67.** (G)** Correlation analysis of ALKBH5 and ABCA1 protein expression levels in immunohistochemistry staining. **(H)** Correlation analysis of ABCA1 and Ki67 protein expression levels in immunohistochemistry staining. **(I)** General observation of lung tissues of the nude mice treated with the indicated cells. **(J)** Counting of pulmonary metastatic nodules at the end.

Afterward, we verified the role of the ALKBH5-m^6^A-ABCA1 axis *in vivo*. We established subcutaneous implantation and tail vein injection nude mouse models with stable ALKBH5 knockdown, double knockdown, and negative control A375 cells. Double knockdown of ALKBH5 and ABCA1 attenuated the inhibition by singly silencing ALKBH5 of melanoma tumor growth in mice reflected by the increase of tumor size and tumor weight (**Figure 7A-7D**). Besides, IHC staining showed that tumors with repressed development caused by ALKBH5 depletion had higher expression of ABCA1 and lower expression of Ki67 than control tumors, and the depletion of ABCA1 alleviated the suppression of Ki67 by ALKBH5 inhibition (**Figure 7E and 7F**). Statistical analysis of IHC staining showed a negative correlation between ALKBH5 and ABCA1 protein expression levels, and a negative correlation between ABCA1 and Ki67 protein levels (**Figure 7G and 7H**). Additionally, we noticed an increase in lung metastasis in the double knockdown A375 cells compared to the single knockdown of ALKBH5 (**Figure 7I and 7J**). The above consequences implied that the loss of ABCA1 alleviated the inhibition on SKCM tumor growth and metastasis by ALKBH5 depletion *in vitro* and *in vivo*.

## Discussion

RNA methylation modification is an important part of epigenetics, in which m^6^A methylation plays the most common modification behavior in mammals and has become an influential field in biomedical research in recent years^10^. Due to its dynamic reversibility, it is considered a critical factor in promoting cancer therapy^12^. Recent studies have proven that m^6^A modification is involved in the occurrence and progression of various tumors^14^. However, whether m^6^A modification plays the role of "pro-cancer" or "anti-cancer" and the underlying mechanisms in human SKCM have not been fully clarified. In a previous report on melanoma, FTO, an m^6^A demethylase, was induced by metabolic starvation stress through the autophagy and NF-κB pathway, promoting melanoma tumorigenesis and anti-PD-1 resistance in melanoma^16^. Moreover, it was stated that m^6^A reader YTHDF3 could affect the metastasis of melanoma and serve as a promising therapeutic target to be interfered with^17^. In our study, we systematically analyzed the expression profiles of key regulators of m^6^A modification in SKCM and found that the m^6^A demethylase ALKBH5, rather than other writers or erasers, was aberrantly overexpressed in human SKCM, simultaneously associated with poorer survival of SKCM patients. We then functionally proved the essential role of ALKBH5 in promoting SKCM growth and metastasis via *in vitro* and *in vivo* assays. Here, we revealed the upregulation of ALKBH5, and it was characterized to be an independent prognostic factor in the SKCM cohort.

ALKBH5, the essential member of m^6^A demethylases, has been reported as a key participant in several malignancies^18^. It is declared that ALKBH5, as an oncogene, enhances macrophage recruitments via the alteration of m^6^A modification of MAP3K8 and promotes hepatocellular carcinoma metastasis^19^. Reversely, ALKBH5 acts as a tumor suppressor gene that represses pancreatic cancer progression by posttranscriptional activation of PER1^20^. Nevertheless, the functions and roles of ALKBH5 in SKCM still need to be clarified. The present study, ALKBH5 was filtered from a cluster of m^6^A regulators according to differential expression and prognostic analysis. We further performed LC-MS analysis to show that the overexpression of ALKBH5 decreases overall m^6^A levels in melanoma cells, indicating that ALKBH5 might affect SKCM progression through m^6^A modification. Through a series of functional assays, we proved that ALKBH5 acted as an oncogene in SKCM via facilitating cell migration, invasion, proliferation, colony formation, and inhibiting autophagy, leading to tumor advancement and metastasis.

To investigate the mechanism of how ALKBH5 promoted SKCM, we employed multi-omics sequencing and regarded ABCA1 as a direct downstream target of ALKBH5-mediated m^6^A demethylation in SKCM, which was upregulated with a better prognosis after ALKBH5 depletion. ABCA1 is a previously reported tumor suppressor gene, translated as transmembrane proteins widely expressed in many tissues, with the most explored function being to assist in cholesterol efflux^21^, ^22^. Cellular cholesterol homeostasis is highly associated with membrane-anchored signaling pathways, altered during tumor cell proliferation^23^. Furthermore, tumor cells have been previously discovered with higher cholesterol levels, indicating an increase in cholesterol metabolism and a lack of cholesterol efflux during cancer development^24^, ^25^. ABCA1-mediated cholesterol efflux is one of the major cholesterol trends, and previous studies have researched the involvement of ABCA1 in tumor development^26^, ^27^. In breast cancer, the MEK/ERK/c-Jun/ABCA1 pathway was activated, leading to cholesterol efflux and membrane fluidity enhancement, thereby promoting the epithelial-mesenchymal transition of cancer cells^28^. Likewise, ABCA1 downregulation caused by ABCA1 promoter hypermethylation led to elevated cholesterol levels in cancer cells, enhanced cell proliferation, and inhibited apoptosis^29^. In addition, a recent article reported that ABCA1 was less expressed in renal clear cell carcinoma tissues than in adjacent tissues, and the overexpression of ABCA1 upstream LXR-α could trigger autophagy and ABCA1-mediated cholesterol efflux, ultimately inhibiting tumor growth^30^. In our study, luciferase assays confirmed the direct binding of ALKBH5 to the m^6^A methylation motif on 3'UTR of ABCA1 mRNA. Meanwhile, MeRIP-qPCR confirmed the increased ABCA1 methylation level after knocking down ALKBH5, suggesting that ALKBH5 directly mediates ABCA1 m^6^A demethylation and leads to abnormal expression. In addition, the results of RNA decay assay showed a decrease in ABCA1 mRNA degradation after silencing ALKBH5. The results indicated that the increased level of m^6^A methylation at the 3'UTR site of ABCA1 may lead to enhanced mRNA stability, which was major responsible for the high expression of ABCA1 membrane protein. Survival analysis indicated that high expression of ABCA1 in SKCM was associated with better prognosis and might exist as a tumor suppressor gene. Subsequent *in vivo* and *in vitro* rescue experiments have shown that ABCA1 can reverse the function of ALKBH5 in promoting the invasiveness of SKCM, which may partially explain how ALKBH5-m^6^A-ABCA1 axis affected the progression and metastasis of SKCM.

Autophagy is a mechanism by which cellular material is degraded for basal turnover of cell components and energy^31^. Programmed cell death is one of the essential methods against cancer, in which autophagy has been playing opposing roles to cancer, and interventions for autophagy have been proposed as cancer therapies^32^-^36^. Previous articles reported that ABCA1-related cholesterol efflux may be accompanied by autophagy and play a therapeutic role in tumors^22^, ^30^. Similarly, in our study, knocking down ALKBH5 increased the expression of ABCA1 and decreased autophagy substrate SQSTM1, which activated autophagy-related pathways for anti-cancer effects. At the same time, the depletion of ABCA1 could elevate SQSTM1 expression, which indicated the repression of autophagy. Recent studies have shown that m^6^A modification mediates regulation of autophagy-related genes, affecting autophagy regulatory network and programmed cell death in multiple diseases^37^, ^38^. Based on the results of our study and previous reports, we may reasonably speculate that the epigenetic modification of ABCA1 mediated by m^6^A demethylase ALKBH5 may affect tumor progression of SKCM via modulating cellular cholesterol homeostasis and autophagy. Nevertheless, this speculation still needs further in-depth validation in the future.

**Figure 8:**
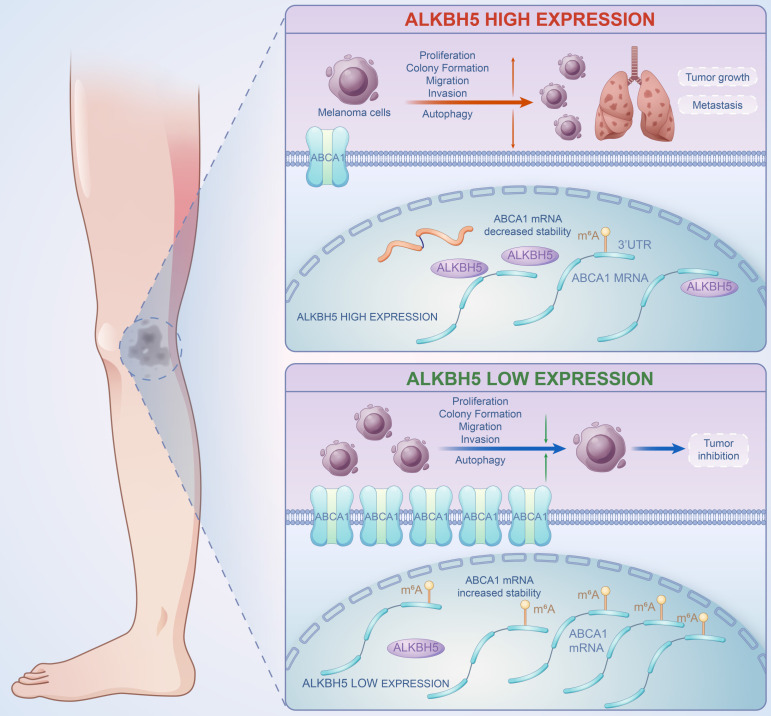
A brief plot summarizing the mechanism of this study. In SKCM, m^6^A demethylase ALKBH5 is highly expressed, binding to the m^6^A motif in the 3'UTR region of ABCA1 mRNA, leading to the demethylation, a reduction in ABCA1 mRNA stability, and a lower expression of ABCA1 membrane protein, causing the increase in cell proliferation, colony formation, migration, invasion ability, and inhibition of autophagy of SKCM cells, ultimately promoting SKCM tumor growth and metastasis.

In summary, our work revealed the functional role of ALKBH5 in regulating the m^6^A methylation modification and the development of SKCM via *in vivo* and *in vitro* assays. Multi-transcriptomics showed that ABCA1 was the downstream target of ALKBH5-mediated epigenetic regulation. Mechanically speaking, ALKBH5 recognized the m^6^A motif on ABCA1 3'UTR, lowering its mRNA stability and protein expression to facilitate tumor advance (**Figure 8**). The ALKBH5-m^6^A-ABCA1 axis could play an essential role in the progression and metastasis of SKCM and might serve as a novel potential therapeutic target for SKCM patients.

## Supplementary Material

Supplementary tables.

## Abbreviations

SKCM: Skin cutaneous melanoma
M^6^A: N^6^-methyladenosine
NS: Normal skin
ALKBH5: Alkylation repair homolog protein 5 ALKBH5
ABCA1: ATP-binding cassette transporter A1
SQSTM1/p62: Sequestosome 1
UTR: Untranslated region
