# Supplementary material for: ALKBH5 facilitates the progression of skin cutaneous melanoma via mediating ABCA1 demethylation and modulating autophagy in an m6A-dependent manner
Source: Int J Biol Sci. 2024 Feb 25;20(5):1729–43. doi: 10.7150/ijbs.92994 (PMC10929202; doi:10.7150/ijbs.92994)
Supplement: Supplementary file 1 — Supplementary tables. [file ijbsv20p1729s1.pdf]

Table S1. Sequences of shRNA.

Gene	shRNA sequences
Scramble	CCTAAGGTTAAGTCGCCCTCG
shALKBH5#1	GAAAGGCTGTTGGCATCAATA
shALKBH5#2	CCTCAGGAAGACAAGATTAGA
shALKBH5#3	GATGAAATCACTCACTGCATA
shABCA1	ACCTATGTGAAACTCTATTAT

Table S2. Primers for RT-qPCR.

Gene	Sequence (5′-3′)
ALKBH5: F	GTGTCCTTCTTTAGCGACTCTGC
ALKBH5: R	GGCCGTATGCAGTGAGTGATTT
ABCA1: F	ACAGCAGTTGGATGGCTTAGATT
ABCA1: R	GCTTGTTCAGGTTGACACACTCC
GAPDH: F	GGAAGCTTGTCATCAATGGAAATC
GAPDH: R	TGATGACCCTTTTGGCTCCC

Table S3. The patient information of SKCM and NS samples collected from First Affiliated Hospital of Sun Yat-sen University.

Inpatient ID	Category	Gender	Age (year)	Location
P20024021	Normal Skin	Female	28	Trunk
P22019663	Normal Skin	Male	29	Trunk
P22021573	Normal Skin	Female	36	Extremities
P22022758	Normal Skin	Male	31	Trunk
P22024141	Normal Skin	Female	35	Trunk
P21004754	Normal Skin	Male	67	Trunk
P20017209	Normal Skin	Female	46	Extremities
P16923	Normal Skin	Male	19	Extremities
N81440	Skin Cutaneous Melanoma	Male	43	Face
P20024941	Skin Cutaneous Melanoma	Male	49	Extremities
P21022858	Skin Cutaneous Melanoma	Male	52	Neck
P22025904	Skin Cutaneous Melanoma	Female	38	Extremities
F20005543	Skin Cutaneous Melanoma	Female	46	Extremities
F20006083	Skin Cutaneous Melanoma	Male	79	Extremities
P20021402	Skin Cutaneous Melanoma	Male	70	Trunk
P09321	Skin Cutaneous Melanoma	Female	58	Extremities
F191794	Skin Cutaneous Melanoma	Male	51	Neck
N8309	Skin Cutaneous Melanoma	Female	63	Extremities
N66990	Skin Cutaneous Melanoma	Male	66	Extremities
F183170	Skin Cutaneous Melanoma	Male	28	Extremities
F171020	Skin Cutaneous Melanoma	Female	68	Face
P21030929	Skin Cutaneous Melanoma	Male	54	Trunk
P21027940	Skin Cutaneous Melanoma	Female	36	Extremities
P21022858	Skin Cutaneous Melanoma	Male	33	Extremities
P20019727	Skin Cutaneous Melanoma	Male	54	Extremities
P22025311	Skin Cutaneous Melanoma	Male	67	Extremities
